# Supplementary material for: Estimating the cost of illness of acute Japanese encephalitis and sequelae care in Vietnam and Laos: A cross-sectional study
Source: PLOS Glob Public Health. 2023 Jun 13;3(6):e0001873. doi: 10.1371/journal.pgph.0001873 (PMC10263309; doi:10.1371/journal.pgph.0001873)
Supplement: S1 Table — (DOCX) [file pgph.0001873.s001.docx]

**Estimating the cost of illness of acute Japanese encephalitis and sequelae care in Vietnam and Laos: A cross sectional study**

# S1 Table. Estimated sample size by stage of illness.

|  | | Acute JE care | Initial rehabilitation and sequelae care | Long-term rehabilitation and sequelae care | Total |
| --- | --- | --- | --- | --- | --- |
| Vietnam | Estimated number in sample frame | 252 | 444 | 1774 |  |
|  | Sample size | 70 | 79 | 91 | **240** |
| Laos | Estimated number in sample frame | 20 | 33 | 131 |  |
|  | Sample size | - | 24 | 55 | **79** |
