# Supplementary material for: Estimating the cost of illness of acute Japanese encephalitis and sequelae care in Vietnam and Laos: A cross-sectional study
Source: PLOS Glob Public Health. 2023 Jun 13;3(6):e0001873. doi: 10.1371/journal.pgph.0001873 (PMC10263309; doi:10.1371/journal.pgph.0001873)
Supplement: Attachment — Submitted filename: JECOI-Response to Reviewers.docx [file pgph.0001873.s004.docx]

Habib Hasan Farooqui, MBBS, MD
Academic Editor
PLOS Global Public Health

March 15, 2023

Dear Dr Farooqui,

We would like to thank the Editors and Reviewers for their time and valuable feedback. Please see below for our responses. Relevant changes to the manuscript have been made as tracked changes.

**Journal Requirements:**

1. You indicated that you had ethical approval for your study. In your Methods section, please ensure you have also stated whether you obtained consent from parents or guardians of the minors included in the study or whether the research ethics committee or IRB specifically waived the need for their consent.

Thank you for your careful review. We have updated the statement on obtained consent in line 226-227 of the all markup, tracked changes version of the revised manuscript, under Methods section – Ethics sub-section.

2. In the online submission form, you indicated that "The data that support the findings of this study are available on request from the corresponding author". All PLOS journals now require all data underlying the findings described in their manuscript to be freely available to other researchers, either 1. In a public repository, 2. Within the manuscript itself, or 3. Uploaded as supplementary information.
This policy applies to all data except where public deposition would breach compliance with the protocol approved by your research ethics board. If your data cannot be made publicly available for ethical or legal reasons (e.g., public availability would compromise patient privacy), please explain your reasons by return email and your exemption request will be escalated to the editor for approval. Your exemption request will be handled independently and will not hold up the peer review process, but will need to be resolved should your manuscript be accepted for publication. One of the Editorial team will then be in touch if there are any issues.

Thank you for your instruction. We are uploading the data files as supplementary information during resubmission process.

**Reviewer 1**

1. What kind of gifts are included in the cost estimation which form part of miscellaneous cost?

We explored any expense for gifts such as fruits, snacks, toys, etc. that household members brought to the patient during hospitalization. However, there was no observation having this type of expense in our records.

2. The amount of total costs in Table 3 & Table 4 are not matching total of other components in the same table. Please explain why these totals are different.

The total costs in Table 3 and Table 4 are not equal to the sum of components’ costs, because each cost value in these tables is the average cost across the subjects who had that type of expense (the denominator is the sample size n indicated firstly in the parenthesis next to the cost value). We included a note on this underneath each table and also indicated in the table captions “…costs for patients who accessed care”.

3. The linkage of the cost calculation and vaccination status is not clearly established. The study focuses mostly on OOP and societal cost of care and conclusion directly linking vaccination with cost is misleading.

The work done by the team is of high standards but the objective of the study and conclusion needs further data and analysis to arrive at the findings. Limiting this study to impact of JE on health system would be advisable rather than linking it directly to vaccine coverage.

Thank you for your recommendation. We have removed the direct linkage of vaccination in the abstract conclusion of the revised manuscript.

The main objective of this study is to estimate the cost of illness to underscore the value of JE prevention. We explored the patients’ JE vaccination status since immunization is the most effective method for prevention and JE vaccines have been introduced in Vietnam and Laos for many years. We included the results and discussions on JE vaccination along the manuscript (in table 2 and lines 237-241, 253-254, 348-356, 368-375, 409-417 in the all markup, tracked changes version of the revised manuscript). Our recommendation on vaccination is based on such findings.

**Reviewer 2**

*General comments*
This is a good paper on a topical issue that may inform policymakers in improving Japanese encephalitis vaccination coverage but may benefit from further review considering the comments below.

*Specific comments*
ABSTRACT
Under the background rearrange the section by mentioning the burden of the disease across the region and state how the aim of the study. Move the perspective of the study to the methodology section.

Thank you for your suggestion. We have moved the perspectives of the study to the methodology section as in the revised manuscript.

Under the methodology section, the author can state the perspective, the unit cost, and the type of cost estimate of the study.

We have rearranged the methodology section to provide information as recommended in the revised manuscript. Methodology section now reads as follows (line 32-37):

*Methodology: We conducted a cross-sectional retrospective study using a micro-costing approach from the health system and household perspectives. Out-of-pocket direct medical and non-medical costs, indirect costs, and family impact were reported by patients and/or caregivers. Hospitalization costs were extracted from hospital charts. Acute costs covered expenditures from pre-hospital to follow-up visits while sequelae care costs were estimated from expenditures in the last 90 days. All costs are in 2021 US dollars.*

Under the results section, it would also be great to present the annual case for initial sequelae care and long-term sequelae care for Laos separately.

We have separated the costs for initial and long-term sequelae care in Laos as in the revised manuscript. It now reads: (line 47-50 in the all markup, tracked changes version of the revised manuscript)

*In Laos, the mean hospitalization costs in acute stage were $2,005 (median $1,698, SE $279) and the mean annual costs were $2,317 (median $0, SE $2,233) for initial sequelae care and $89 (median $0, SE $57) for long-term sequelae care.*

INTRODUCTION
To show the burden of care by households and the overall health financing system. The research could provide the percentage of out-of-pocket and health insurance expenditures in Laos from previous studies.

We have added the following text with reference to include the recommended information: (line 115-118 in the all markup, tracked changes version of the revised manuscript)

*A major source of healthcare financing in Laos is out-of-pocket expenditure. Even with national health insurance, patients still need a co-pay of 25% if the treatment has high cost (exceeding $600). As such, understanding the cost of the illness emphasizes the need to prevent JE infection and avoid potential catastrophic expenditure. (18)*

METHODS
It would be great to include a 'unit' cost typology to summarize the resource use measurement of the study, for example, including intervention 'unit' costs, direct and ancillary service 'unit' costs, activity costs, and input costs in the topology.

The analysis section discusses the unit costs for medical costs charged by providers, which were assumed to cover overhead costs. The resource use measurement and the methods used to capture the quantities of resources need to be clarified having applied the micro-costing approach. The research could consider providing a resource measurement section.

Thank you for your recommendation. We have added the text in Methods section (line 195-196 in the all markup, tracked changes version of the revised manuscript) and a column to describe unit of each cost item in Table 1 in the revised manuscript.

RESULTS
The results section only shows detailed results of Vietnam's direct non-medical costs for patients who access care in Vietnam and fails to present the breakdown costs for Laos. The researcher could provide a summarized cost for the different health perspectives, i.e., the health system and the household, as per the study perspective. This will enable easy comparison and also present the results per the study's main aim.

We have added Table 5 to present breakdown of costs for patients who access care in Laos. (line 325-328 in the all markup, tracked changes version of the revised manuscript)

DISCUSSION
How could the study be compared to other regional studies, especially on the cost of implementing vaccination programmes? The researcher could consider referring to a cost-effectiveness study done on the JEVAX to affirm the conclusion of expanding the vaccine coverage.

Thank you for your great suggestion. We have added the following text with citations into the revised manuscript (line 368-375 in the all markup, tracked changes version of the revised manuscript)

*Our estimation on the direct medical cost of care for acute JE and sequelae is comparable to treatment costs reported in cost-effectiveness study of JE vaccination in Shanghai and Guizhou, China (27,28); but higher than the measures in Cambodia which had a possibility of underestimation as long-term cost was not extrapolated beyond a three-month period (26) and in Bali, Indonesia where treatment costs for JE-associated disability were based on experts’ opinion.(29) Our findings thus can expand the evidence to inform JE vaccination cost-effectiveness, which was found in previous studies as cost-saving or highly cost-effective with similar or lower cost of illness.(26–30)*
